# Methyl 4-methyl­benzoate

**DOI:** 10.1107/S1600536808008738

**Published:** 2008-04-10

**Authors:** Aamer Saeed, Hummera Rafique, Ulrich Flörke

**Affiliations:** a Department of Chemistry, Quaid-i-Azam University Islamabad, Pakistan; b Department Chemie, Fakultät für Naturwissenschaften, Universität Paderborn, Warburgerstrasse 100, D-33098 Paderborn, Germany

## Abstract

The structure of the title compound, C_9_H_10_O_2_, is related to that of 4-methyl­phenyl 4-methyl­benzoate and ethyl­ene di-4-methyl­benzoate showing similar bond parameters. The mol­ecule is planar, the dihedral angle between the aromatic ring and the –COOMe group being 0.95 (6)°. The cystal structure exhibits inter­molecular C—H⋯O contacts that link mol­ecules into infinite chains extended in the [001] direction.

## Related literature

For related literature, see: Deguire & Brisse (1988 ▶); Gowda *et al.* (2007 ▶; Gray & Whalley (1971 ▶); Harris & Mantle (2001 ▶); Saeed & Rama (1994 ▶); Simpson (1978 ▶).
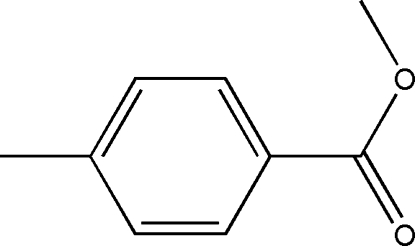


## Experimental

### 

#### Crystal data

C_9_H_10_O_2_
*M* *_r_* = 150.17
Monoclinic, 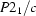

*a* = 5.9134 (11) Å
*b* = 7.6048 (14) Å
*c* = 17.484 (3) Å
β = 97.783 (4)°
*V* = 779.0 (2) Å^3^
*Z* = 4
Mo *K*α radiation
μ = 0.09 mm^−1^
*T* = 120 (2) K
0.45 × 0.43 × 0.39 mm

#### Data collection

Bruker SMART APEX diffractometer
Absorption correction: multi-scan (*SADABS*; Sheldrick, 2004 ▶) *T*_min_ = 0.961, *T*_max_ = 0.967
6617 measured reflections
1855 independent reflections
1482 reflections with *I* > 2σ(*I*)
*R*_int_ = 0.038

#### Refinement

*R*[*F* ^2^ > 2σ(*F* ^2^)] = 0.042
*wR*(*F* ^2^) = 0.124
*S* = 1.06
1855 reflections
102 parameters
H-atom parameters constrained
Δρ_max_ = 0.31 e Å^−3^
Δρ_min_ = −0.20 e Å^−3^

### 

Data collection: *SMART* (Bruker, 2002 ▶); cell refinement: *SAINT* (Bruker, 2002 ▶); data reduction: *SAINT*; program(s) used to solve structure: *SHELXS97* (Sheldrick, 2008 ▶); program(s) used to refine structure: *SHELXL97* (Sheldrick, 2008 ▶); molecular graphics: *SHELXTL* (Sheldrick, 2008 ▶); software used to prepare material for publication: *SHELXTL*.

## Supplementary Material

Crystal structure: contains datablocks I, global. DOI: 10.1107/S1600536808008738/sg2231sup1.cif

Structure factors: contains datablocks I. DOI: 10.1107/S1600536808008738/sg2231Isup2.hkl

Additional supplementary materials:  crystallographic information; 3D view; checkCIF report

## Figures and Tables

**Table 1:** Hydrogen-bond geometry (Å, °)

*D*—H⋯*A*	*D*—H	H⋯*A*	*D*⋯*A*	*D*—H⋯*A*
C9—H9*B*⋯O2^i^	0.98	2.51	3.4930 (16)	177

Symmetry code: (i) 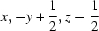.

**Fig. 1.**
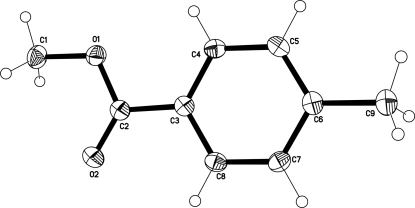
Molecular structure of title compound. Displacement ellipsoids are drawn at the 50% probability level.

**Fig. 2.**
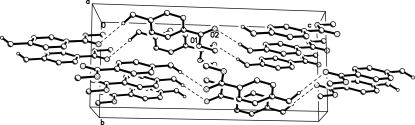
Crystal packing viewed along [100] with intermolecular hydrogen bonding pattern indicated as dashed lines. H-atoms not involved in hydrogen bonding are omitted.

## supplementary crystallographic information

### Comment

The title ester is an important intermediate in the synthesis of a variety of natural products. These include the sclerotiorin group of fungal metabolites (Gray & Whalley, 1971), isochromans related to sclerotiorin pigments (Saeed & Rama, 1994) and isocoumarins like the 7-methylmellein (Harris & Mantle, 2001) and stellatin (Simpson, 1978).

### Experimental

The title ester was prepared from commercial *p*-toluic acid according to standard procedure.

### Refinement

Hydrogen atoms were located in difference syntheses, refined at idealized positions riding on the carbon or nitrogen atoms (C–H = 0.88–0.99 Å) with isotropic displacement parameters *U*_iso_(H) = 1.2U(C_eq_).

### Crystal data

**Table:**

C_9_H_10_O_2_	*F*_000_ = 320
*M**_r_* = 150.17	*D*_x_ = 1.280 Mg m^−^^3^
Monoclinic, *P*2_1_/*c*	Mo *K*α radiation λ = 0.71073 Å
Hall symbol: -P 2ybc	Cell parameters from 806 reflections
*a* = 5.9134 (11) Å	θ = 2.4–27.8º
*b* = 7.6048 (14) Å	µ = 0.09 mm^−^^1^
*c* = 17.484 (3) Å	*T* = 120 (2) K
β = 97.783 (4)º	Block, colourless
*V* = 779.0 (2) Å^3^	0.45 × 0.43 × 0.39 mm
*Z* = 4	

### Data collection

**Table:**

Bruker SMART APEX diffractometer	1855 independent reflections
Radiation source: sealed tube	1482 reflections with *I* > 2σ(*I*)
Monochromator: graphite	*R*_int_ = 0.039
*T* = 120(2) K	θ_max_ = 27.9º
φ and ω scans	θ_min_ = 2.4º
Absorption correction: multi-scan(SADABS; Sheldrick, 2004)	*h* = −7→7
*T*_min_ = 0.961, *T*_max_ = 0.967	*k* = −10→9
6617 measured reflections	*l* = −23→23

### Refinement

**Table:**

Refinement on *F*^2^	Secondary atom site location: difference Fourier map
Least-squares matrix: full	Hydrogen site location: difference Fourier map
*R*[*F*^2^ > 2σ(*F*^2^)] = 0.042	H-atom parameters constrained
*wR*(*F*^2^) = 0.124	*w* = 1/[σ^2^(*F*_o_^2^) + (0.0752*P*)^2^ + 0.0208*P*] where *P* = (*F*_o_^2^ + 2*F*_c_^2^)/3
*S* = 1.06	(Δ/σ)_max_ < 0.001
1855 reflections	Δρ_max_ = 0.31 e Å^−^^3^
102 parameters	Δρ_min_ = −0.20 e Å^−^^3^
Primary atom site location: structure-invariant direct methods	Extinction correction: none

### Special details

**Table:**

Geometry. All e.s.d.'s (except the e.s.d. in the dihedral angle between two l.s. planes) are estimated using the full covariance matrix. The cell e.s.d.'s are taken into account individually in the estimation of e.s.d.'s in distances, angles and torsion angles; correlations between e.s.d.'s in cell parameters are only used when they are defined by crystal symmetry. An approximate (isotropic) treatment of cell e.s.d.'s is used for estimating e.s.d.'s involving l.s. planes.
Refinement. Refinement of *F*^2^ against ALL reflections. The weighted *R*-factor *wR* and goodness of fit *S* are based on *F*^2^, conventional *R*-factors *R* are based on *F*, with *F* set to zero for negative *F*^2^. The threshold expression of *F*^2^ > σ(*F*^2^) is used only for calculating *R*-factors(gt) *etc*. and is not relevant to the choice of reflections for refinement. *R*-factors based on *F*^2^ are statistically about twice as large as those based on *F*, and *R*- factors based on ALL data will be even larger.

### Fractional atomic coordinates and isotropic or equivalent isotropic displacement parameters (Å^2^)

**Table:**

	*x*	*y*	*z*	*U*_iso_*/*U*_eq_	
O1	0.39091 (14)	0.28701 (11)	0.44793 (5)	0.0280 (2)	
O2	0.68910 (15)	0.15425 (13)	0.51751 (5)	0.0325 (3)	
C1	0.2956 (2)	0.31740 (17)	0.51874 (7)	0.0320 (3)	
H1A	0.2793	0.2050	0.5448	0.048*	
H1B	0.1456	0.3732	0.5068	0.048*	
H1C	0.3974	0.3946	0.5526	0.048*	
C2	0.59091 (19)	0.20144 (15)	0.45593 (6)	0.0234 (3)	
C3	0.67753 (18)	0.17434 (15)	0.38071 (6)	0.0223 (3)	
C4	0.55841 (19)	0.23124 (15)	0.31083 (7)	0.0247 (3)	
H4A	0.4154	0.2888	0.3098	0.030*	
C5	0.6496 (2)	0.20350 (15)	0.24261 (7)	0.0262 (3)	
H5A	0.5675	0.2426	0.1952	0.031*	
C6	0.8588 (2)	0.11953 (15)	0.24239 (7)	0.0244 (3)	
C7	0.97615 (19)	0.06389 (15)	0.31291 (7)	0.0253 (3)	
H7A	1.1195	0.0068	0.3140	0.030*	
C8	0.88716 (19)	0.09050 (15)	0.38126 (7)	0.0242 (3)	
H8A	0.9693	0.0515	0.4287	0.029*	
C9	0.9593 (2)	0.08897 (17)	0.16858 (7)	0.0312 (3)	
H9A	1.1213	0.1213	0.1764	0.047*	
H9B	0.8782	0.1613	0.1273	0.047*	
H9C	0.9438	−0.0355	0.1542	0.047*	

### Atomic displacement parameters (Å^2^)

**Table:**

	*U*^11^	*U*^22^	*U*^33^	*U*^12^	*U*^13^	*U*^23^
O1	0.0268 (5)	0.0322 (5)	0.0260 (4)	0.0042 (3)	0.0071 (3)	0.0018 (3)
O2	0.0343 (5)	0.0389 (5)	0.0234 (5)	0.0042 (4)	0.0004 (4)	0.0016 (3)
C1	0.0340 (7)	0.0342 (7)	0.0299 (7)	0.0024 (5)	0.0121 (5)	−0.0009 (5)
C2	0.0249 (6)	0.0201 (6)	0.0248 (6)	−0.0033 (4)	0.0022 (5)	0.0003 (4)
C3	0.0236 (6)	0.0203 (6)	0.0232 (6)	−0.0031 (4)	0.0031 (4)	0.0006 (4)
C4	0.0214 (5)	0.0252 (6)	0.0271 (6)	0.0008 (4)	0.0022 (4)	0.0024 (4)
C5	0.0271 (6)	0.0281 (6)	0.0222 (6)	−0.0019 (5)	−0.0006 (5)	0.0029 (4)
C6	0.0275 (6)	0.0210 (6)	0.0251 (6)	−0.0058 (4)	0.0049 (4)	−0.0010 (4)
C7	0.0231 (6)	0.0215 (6)	0.0316 (6)	0.0008 (4)	0.0042 (5)	0.0002 (4)
C8	0.0245 (6)	0.0221 (6)	0.0251 (6)	−0.0017 (4)	0.0000 (4)	0.0032 (4)
C9	0.0355 (7)	0.0320 (7)	0.0271 (6)	−0.0005 (5)	0.0079 (5)	−0.0018 (5)

### Geometric parameters (Å, °)

**Table:**

O1—C2	1.3405 (14)	C5—C6	1.3927 (17)
O1—C1	1.4468 (14)	C5—H5A	0.9500
O2—C2	1.2065 (14)	C6—C7	1.3962 (17)
C1—H1A	0.9800	C6—C9	1.5101 (16)
C1—H1B	0.9800	C7—C8	1.3843 (16)
C1—H1C	0.9800	C7—H7A	0.9500
C2—C3	1.4890 (16)	C8—H8A	0.9500
C3—C8	1.3929 (16)	C9—H9A	0.9800
C3—C4	1.3940 (16)	C9—H9B	0.9800
C4—C5	1.3899 (16)	C9—H9C	0.9800
C4—H4A	0.9500		
			
C2—O1—C1	115.38 (9)	C4—C5—H5A	119.3
O1—C1—H1A	109.5	C6—C5—H5A	119.3
O1—C1—H1B	109.5	C5—C6—C7	118.16 (10)
H1A—C1—H1B	109.5	C5—C6—C9	121.71 (11)
O1—C1—H1C	109.5	C7—C6—C9	120.13 (11)
H1A—C1—H1C	109.5	C8—C7—C6	121.10 (10)
H1B—C1—H1C	109.5	C8—C7—H7A	119.5
O2—C2—O1	123.28 (10)	C6—C7—H7A	119.5
O2—C2—C3	124.43 (11)	C7—C8—C3	120.20 (10)
O1—C2—C3	112.28 (9)	C7—C8—H8A	119.9
C8—C3—C4	119.46 (10)	C3—C8—H8A	119.9
C8—C3—C2	118.00 (10)	C6—C9—H9A	109.5
C4—C3—C2	122.54 (10)	C6—C9—H9B	109.5
C5—C4—C3	119.76 (11)	H9A—C9—H9B	109.5
C5—C4—H4A	120.1	C6—C9—H9C	109.5
C3—C4—H4A	120.1	H9A—C9—H9C	109.5
C4—C5—C6	121.33 (10)	H9B—C9—H9C	109.5
			
C1—O1—C2—O2	−1.07 (16)	C3—C4—C5—C6	0.00 (17)
C1—O1—C2—C3	179.72 (9)	C4—C5—C6—C7	−0.20 (17)
O2—C2—C3—C8	−0.70 (18)	C4—C5—C6—C9	−179.94 (10)
O1—C2—C3—C8	178.50 (10)	C5—C6—C7—C8	0.28 (17)
O2—C2—C3—C4	−179.94 (11)	C9—C6—C7—C8	−179.98 (10)
O1—C2—C3—C4	−0.74 (16)	C6—C7—C8—C3	−0.16 (17)
C8—C3—C4—C5	0.12 (17)	C4—C3—C8—C7	−0.05 (17)
C2—C3—C4—C5	179.36 (10)	C2—C3—C8—C7	−179.32 (10)

### Hydrogen-bond geometry (Å, °)

**Table:**

*D*—H···*A*	*D*—H	H···*A*	*D*···*A*	*D*—H···*A*
C9—H9B···O2^i^	0.98	2.51	3.4930 (16)	177

Symmetry codes: (i) *x*, −*y*+1/2, *z*−1/2.
